# Simplified modeling of *E. coli* mortality after genome damage induced by UV-C light exposure

**DOI:** 10.1038/s41598-020-67838-1

**Published:** 2020-07-09

**Authors:** Jaime Sánchez-Navarrete, Nancy Jannet Ruiz-Pérez, Armando Guerra-Trejo, Julia Dolores Toscano-Garibay

**Affiliations:** 1 Laboratorio de Investigación en Toxicología, Hospital Juárez de México, Av. Instituto Politécnico Nacional #5160 Col. Magdalena de Las Salinas, Ciudad de México, Mexico, C.P. 07760 Mexico; 2 Departamento de Biofísica, Escuela Nacional de Ciencias Biológicas, Instituto Politécnico Nacional, Prolongación de Carpio Y Plan de Ayala S/N, Col. Santo Tomás, Ciudad de México, Mexico, C.P. 11340 Mexico

**Keywords:** Biophysics, Microbiology, Physics

## Abstract

UV light is a group of high-energy waves from the electromagnetic spectrum. There are three types of UV radiations: UV-A, -B and -C. UV-C light are the highest in energy, but most are retained by the ozone layer. UV-A and -B reach the earth’s surface and cause damage on living organisms, being considered as mutagenic physical agents. Numerous test models are used to study UV mutagenicity; some include special lamps, cell cultures and mathematical modeling. Mercury lamps are affordable and useful sources of UV-C light due to their emission at near the maximum absorption peak of nucleic acids. *E. coli* cultures are widely used because they have DNA-damage and -repairing mechanisms fairly similar to humans. In here we present two simple models that describe UV-C light incidence on a genome matrix, using fundamental quantum–mechanical concepts and considering light as a particle with a discontinuous distribution. To test the accuracy of our equations, stationary phase cultures of several *E. coli* strains were exposed to UV-C light in 30 s-intervals. Surviving CFUs were counted and survival/mortality curves were constructed. These graphs adjusted with high goodness of fit to the regression predictions. Results were also analyzed using three main parameters: quantum yield, specific speed and time of mortality.

## Introduction

The main source of energy of our planet is the Sun. The nuclear fusion occurring within its core follows a fixed proton-proton chain reaction known as the Critchfield's cycle that ultimately results in light and energy dispersed in a wide eletromagnetic spectrum ([p^+^  + p^+^ → H_2_ + e^+^  + *Ʋ*_*e*_, H_2_ + p^+^ → He_3_ + *gamma, visible or UV*];[He_3_ + He_3_ → He_4_ + p^+^  + p^+^])^1^.

Ultraviolet light (UV light) is the portion of such spectrum constituted by high-energy wavelengths, classified just below ionizing radiations. There are three main UV light regions: type A (UV-A) (315–400 nm), type-B (UV-B) (280–315 nm) and type-C (UV-C) (200–280 nm)^2^. UV-C light is the highest in energy and the more dangerous to living organisms, nonetheless most of it is retained by the ozone layer (OL) in the stratosphere along with other radiations such as cosmic-, gamma- and X-rays and normally do not reach the surface^3–6^. Sun-emited light that hits our planet is made up of visible (62.7%), infrared (31.9%), UV-A (5.1%) and UV-B (0.3%) lights^7^. Biological damages during sun exposure are caused by UV-B, hence is considered the main mutagenic physical agent on earth^8,9^.

Since the 1970’s, NASA has monitored the changes in the OL-thickness observing a gradual but controlled deterioration, particularly at the Antarctica, and an elevation in the frequency of ozone depletion events (mini-holes) on both hemispheres^10–13^. This phenomenon is hindering OL protective capacity against radiations and consequently increasing the probability of high-energy UV light exposure, which might be an important factor contributing to the occurrence of cancer-associated mutations in humans (photocarcinogenesis)^8,9^.

Multiple experimental designs are available to describe and predict the biological effects of UV light. Some of the simpler test systems include the use of artificial light sources inciding on cell cultures and the mathematical modeling of the survival/mortality kinetics.

The most affordable and widely distributed artificial UV light sources are the lamps of low or medium pressure made up of quartz tubes with tungsten electrodes and an atmosphere of mercury/argon. The electric arc produced through such steam, generates light wavelength of 253.7 nm for low-pressure and 240–270 nm for medium-pressure lamps, which are close to the maximum absorption peak of nucleic acids (260 nm)^14^. This wavelength also coincides with the maximum growth inactivation peak in *E. coli* (268 nm) that makes mercury lamps excellent germicides and also a useful tool for the study of UV-provoked biological damage in this bacteria^15^.

Specifically, genomic effects of UV-C light exposure occur through different mechanisms, the most important are by transference of charge through endogenous chromophores to DNA modifying molecules^16^ or by direct absorption of photons by nitrogenous bases^17^. The main DNA lesions by direct absorption are those where photons are strongly acquired by adjacent pyrimidines, which eventually form dimers. There are two main types of dimerization products: 5–6-*cis*-syn-cyclobutane (CPD) and cross-adducts of pyrimidine-6–4 pyrimidones (6-4PP)^18–20^. Pyrimidic dimerization halts DNA replication by interfering with polymerases normal gliding and consequently cell reproduction stops (a process called *inactivation*). In *E. coli*, the formation of thymine-thymine, thymine-cytosine, and/or cytosine-cytosine dimers have a frequency of 59%, 34% and 7%, respectively. Nonetheless, there are several natural mechanisms of dimer-repairment that lead to reactivation of *E. coli* cells, from those that restore DNA under visible light (photoreactivation) to the prone to error SOS system. The nucleotide excision repair mechanism (NER), also referred as dark repair of the SOS response, is a pathway predominantly repairing dsDNA damaged by UV-emissions and it is driven by the UvrABC endonuclease multienzyme complex. NER starts when a complex of two UvrA and one UvrB subunits (A2B) scans DNA in search of injured sites. Then, the UvrA dimer is released and UvrB recruits UvrC that in turn hydrolyses specific phosphodiester bonds around the lession. UvrD helicase then sets loose a 12–13 nt fragment leaving a gap that is finally filled by the DNA polymerase Pol I.

Although this mechanism has been extensively described in bacteria, the process is universal. In humans, NER is conducted by a much larger number of proteins that essentially perform the same steps as UvrABC in *E. coli*, but the difference relies only in the length of the fragment released from the DNA filament (27–29 nt)^21–23^. Hence, *E. coli* cultures under a luminous stimulus are a good test system for the determination of UV light-induced DNA damage, for the observation of cellular repairing capacities and consequently for the description of survival and/or mortality behaviors of cells.

Finally, several mathematical models have been developed that addressed different aspects to describe the dynamics of the aforementioned phenomenon. Some authors consider a wave-like conduct and others a particle nature of UV-C light, however, there are notable differences between each behaviour of light and the damage caused to the cell interior. For instance, propagation of UV light through a cell matrix could be considered as continuous when is thought as a wave, while it should be reckon that DNA bases would discontinuously absorb impacting photons when UV light is visualized as a corpuscular flow.

On the other hand, models such as the Chick-Watson model, consider a single impact stoichiometry for UV light inciding on DNA, that is to say, one photon is sufficient to carry out a photochemical reaction^24^. However, these models do not fully explain the entire cell inactivation processes^23^. Instead, Adair-Aguiar et al. had consider a multiple impact kinetics, where is necessary that two or more photons hit on the same site of a thymine molecule in order to cause a dimerization reaction^25^. *Jagger and Harm*, on the other hand ponder that a number of photons in the order of 10^4^ is required to generate thymine dimers as result of a photoreaction^26^.

Most of the mathematical models are complex descriptions that assume indistinctly a wave/corpuscular behavior of UV light and use multiple hard-to-measure parameters, making them impractical for further applications.

The aim of this work was to develop two simple models to describe and predict the kinetics of viability and mortality of genome damage induced by UV-C light on *E. coli*, considering both: a multiple impact and a corpurscular behavior of light. We used a set of five strains that were differentially mutated as the experimental system; taking advantage of its fast-growing capability, its well-known mutation and DNA repairment mechanisms and the fact that its genomic structure is described in detail^27^. Our mathematical models are of deductive and mechanistic nature; both are based on fundamental quantum concepts and the results were analyzed through three main variables: quantum mortality yield, minimum time of mortality and specific speed of mortality.

## Materials and methods

### Physical and mathematical modeling

#### General assumptions

Models were constructed using differencial and integral calculus, mean value theorem, regression method by least squares and determining fiducial limits. We made the following assumptions:

1. According to quantum theory, UV light has a corpuscular behavior.
2. Dissemination of the total energy of the incident light is discontinuous in a genomic matrix.
3. The energy of two or more photons is absorbed by one base. The energy of one photon could not be enough, but two or more would break/form a molecular bond (multiple impact).
4. The physical model was based on the Planck’s equation, where the energy of each photon is conversely proportional to the wavelength of the incident light (E = n_0_hc/λ). Energy is expressed in einsteins, and n_0_ corresponds to the Avogadro’s constant.
5. Pyrimidine dimers are the major and most important photochemical products on nucleic acids. Purine bases are less sensitive to UV-C light^12,13, 23,28^.
6. Both models were based on the relationship between a specific speed of mortality (SSM) and the energy absorbed in a particular exposure time.
7. We considered a hypothesis, named the *proportionality hypothesis,* were the number of base pairs of a genome is proportional to the lethal impact number (*LIN*).

We used several variables for the mathematical modeling including the effective impact section (EIS), molecular excitation time, frequency of impact, lethal impact number (LIN), number of bases by unit of genomic volume (N*), lethal radiation dosage at 50% (LRD_50_), absolute death state, minimum mortality time (MMT), specific speed of mortality (SSM), and cell quantum mortality yield (cQMY). The genomic volume for each *E. coli* strain was also calculated using LIN and cQMY. The fitness of the developed equations was challenged on the laboratory.

### Experimental procedures

Table 1 enlists the strains of *E. coli* used for the biological experiments. Each strain was selected with specific mutations affecting repairing enzymes.

**Table 1:** *E. Coli* strains with genotypes.

*E.coli* Strain	Genotype	Phenotype	Reference
HfrH180	*(K12 hfr*+*)*	Complete repairing systems	Lavoie and Mathieu^29^, Trudel et al. ^30^
W3110	*F*^*−*^* λ*^*−*^* rph1, IN(rrnD, rrnE)*	Slow pyrimidine de novo synthethic pathway	Bachmann^31^
ATCC25922	Clinical isolate	Complete repairing systems	Bergey’s manual^32^
polA^+^	*W3110 thy*^*−*^* thi*^*−*^* PolA1*^+^	Expression of polA polymerase	Espinosa-Aguirre et al.^33^
polA^-^	*P3478 thy*^*−*^* thi*^*−*^* PolA*^*−*^	Lack of polA polymerase	Espinosa-Aguirre et al.^33^
DH5αthy^-^	*recA*^*−*^* thy*^*−*^	Lack of recA	Manrique-Suárez et al.^34^

Strain DH5αthy^−^ was derived from DH5α mutagenized with methyl-*N*′nitro-*N*-nitrosoguanidine (MNNG, Sigma chemical CO, St Louis MO, USA) according to Manrique-Suarez et al.

### Treatment of* E. coli* strains

Each strain was grown during 18 h in 50 ml of nutrient broth No. 2 (Cat. No. CM0067, OXOID) until density reached a turbidity equivalent to a 0.5 McFarland standard or ≈ 10^8^ cells. Then, 3 ml of each culture were directly poured on dry Petri dishes (area 75.42 cm^2^) and were exposed to UV-C light using a low-pressure mercury lamp (TUV, input voltage 15 W and output 3 W, Cat. No. G15T8, PHILLIPS, Holland) with 253.7 nm in wavelength that was located at a fixed distance of 33 cm, hence a fluence of 2.75 mJ/s cm^2^. Lamp was pre-heated for 10 min before use. Ten dishes of each strain culture were exposed for different times, in 30 s-intervals, up to a maximum exposure lapse of 300 s (fluence: 83–830 mJ/cm^2^, dosage range: 0.627–6.27 J/Petri dish). Subsequently, 500 µl of the content of each dish were collected, serially diluted on saline solution (five dilutions on a decimal scale). Final dilution was mixed with soft agar and spread over nutrient agar plates (Cat.No. OX-CM0003B, OXOID) before incubation at 37 °C during 24 h. Control were obtained as experimental mock-ups. Briefly, 3 ml of the initial stationary culture were poured on dry plates, recovered, serially diluted and cultured under the same conditions as the experimental samples. The number of colony forming units per milliliter (CFU/ml) was determined with a colony counter (Accu-Lite Colony Counter Model 133–8,002, FISHER) and accounting the dilution grade. The number of CFUs from unexposed cultures was considered as 100% of viability. Three experiments were executed by triplicate for each exposure time.

### Statistical analysis

Data on viable counts were processed using a linear regression analysis and calculating the correlation and determination coefficients, fiducial limits, confidence intervals, and finally subjected to a t-Student test by one mean. We report mean values of data from each cultured strain.

## Results

### Construction of the models

#### Physical model

**Figure 1:**
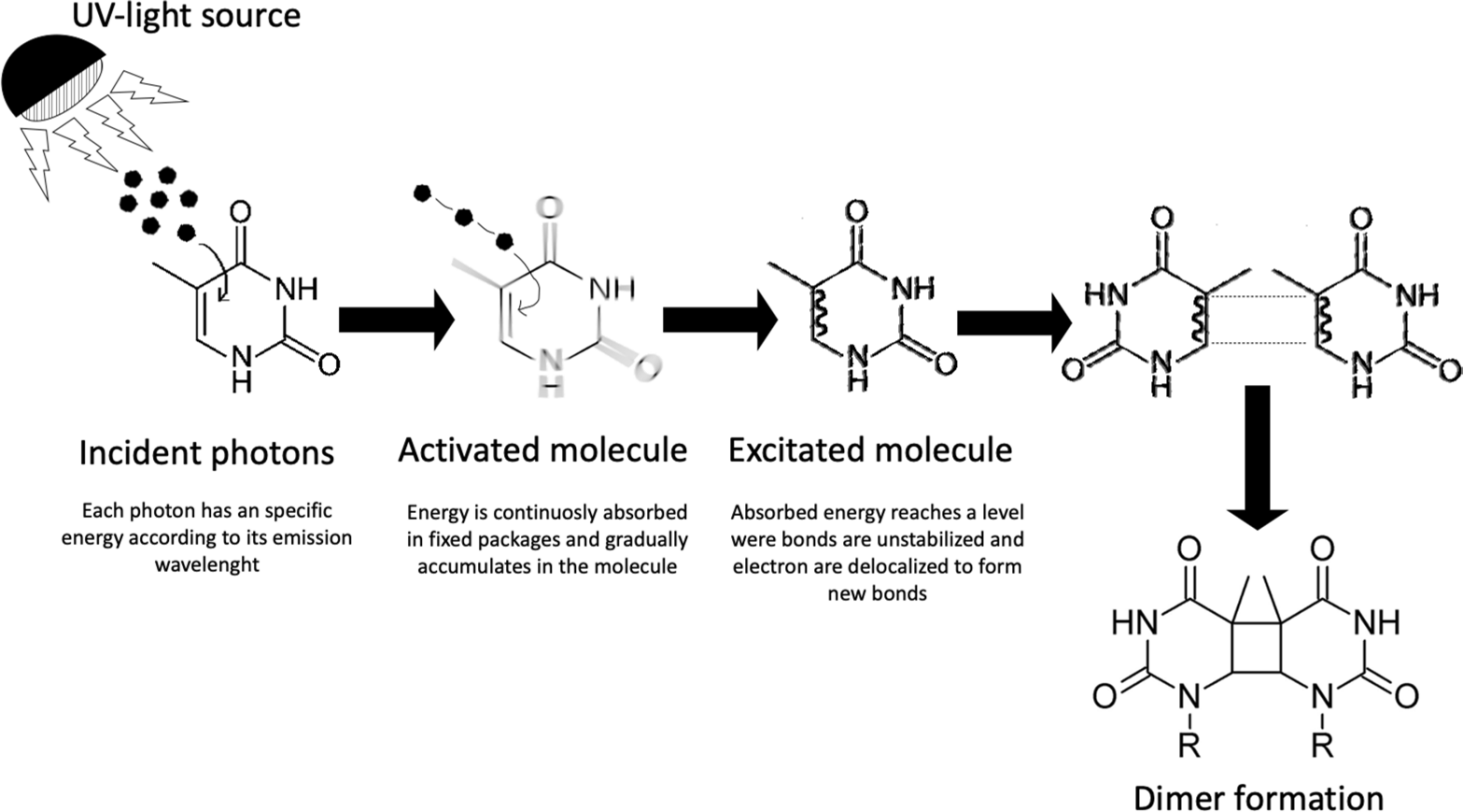
Schematics of basic concepts for the physical model.

Our physical model assumes that the occurrence of photoreactions depend on the number of photons impacting each nitrogenous base, and hence, on the energetic content of the radiation^25,28,35^. From the quantum perspective is expected, first, that an “activation” of the genome occurs when hitted by a luminous radiation, meaning that part of the incident energy has been absorbed but that is still not enough to produce bond breakage. Second, that the energy of the activated molecule increases as the wavelength diminishes; and third, that an activated molecule absorbs a fixed number of quanta to become an excited molecule where energy is suffice to provoke the dislocation of electrons (Fig. 1). If each excited molecule then results in a product molecule, the yield of such reaction will have values under one because it is a multiquantum process, therefore, the absorbed light influences the rate of cell death in a wavelength-dependent manner^36,37^. According to this, for any genomic matrix undergoing a photochemical reaction, each molecule accepts luminous energy independently instead of being evenly distributed among the whole bases.

We defined mortality as the condition were a cell is non-reproductive and has no metabolic activity and the cell quantum mortality yield, or cQMY (${\Phi }_{\upmu }$), as the capacity of quanta with a particular wavelength to cause chemical changes in sensitive bases. In a condition where the stimulus is continuous, each dead cell is the result of the sum of the alterations in the bases (or number of pyrimidine dimers), so cQMY can be expressed in terms of absorbed Einsteins as^37^:

$${\Phi }_{{\upmu }} = \frac{{{\text{Number}}\;{\text{of}}\;{\text{dead}}\;{\text{cells (N)}}\;{\text{on}}\;{\text{a}}\;{\text{given}}\;{\text{time}}\;{\text{(t)}}}}{{{\text{ Number}}\;{\text{of}}\;{\text{absorbed}}\;{\text{einsteins}}\;{\text{on}}\;{\text{the}}\;{\text{same}}\;{\text{time }}}}$$

According to this, the rate of cell death or specific speed of mortality (SSM or $$k = dN/dt)$$ would be proportional to the activation energy or radiation dosage:

$$k \propto \frac{{{\Delta }E}}{t}\;{\text{or}}\;\frac{dN}{{dt}} \propto \frac{{{\Delta }E}}{t}$$

When these expressions are multiplied by a proportionality constant and rearranged, a first order and first grade differential equation is obtained:

1 $$\frac{{{\text{dN}}}}{{{\text{dt}}}} - \Phi _{{\upmu }} \left( {\Delta {\text{E}}/{\text{t}}} \right) = 0\;{\text{or}}\;\Phi _{{\upmu }} = \frac{{\frac{{{\text{dN}}}}{{{\text{dt}}}}}}{{\Delta {\text{E}}/{\text{t}}}}$$

also:

2 $$\frac{{{\text{dN}}}}{{{\text{dt}}}}{\text{ = }}\Phi _{{\upmu }} \left( {\Delta {\text{E}}/{\text{t}}} \right)$$

cQMY is actually the proportionality factor defined as the efficiency by which a luminous stimulus changes the cellular mortality. $\frac{{{\text{dN}}}}{{{\text{dt}}}}$ corresponds to SSM, *ΔE* is the activation energy (also *E*_*a*_), *t* is the exposure time and *N* is the number of dead cells*.* In other words, cQMY corresponds to the number of dead cells due to the formation of dimers in a time unit divided by the number of absorbed Einsteins.

Integrating Eq. (2) and assuming that Ф_μ_ is an unchanging and cell-specific value, we have the following mortality expression:

3 $${\text{ N}} = {\Phi }_{{\upmu }} {\text{E}}_{{\text{a}}} \ln \left( {\text{t}} \right)$$

Considering that after UV light exposure, the number of initial cells (N_0_) is given by the sum of dead plus surviving cells (N_1_), then N_0_ = N + N_1_ and survival is defined by the equation:

4 $${\text{ N}}_{1} = - {\Phi }_{{\upmu }} {\text{E}}_{{\text{a}}} \ln \left( {\text{t}} \right) + {\text{N}}_{0}$$

where N_1_ < N_0_ is always true. From here, *E*_*a*_ can be expressed in terms of wavelength (*λ*) accordingly with assumption 4*,* so:

4a $${\text{N}}_{1} = - \frac{{{\Phi }_{{\upmu }} {\text{n}}_{0} {\text{hc}}}}{{\uplambda }}\ln \left( {\text{t}} \right) + {\text{N}}_{0}$$

The latter equation shows that specific speed of mortality by UV-C light is a converse function of the wavelength and is directly related to the total activation energy.

Besides, depending on cQMY, the time for cell inactivation depends on the nature of the sensitive chemical groups on the genome, its base distribution, sequence of sensitive sites and the functionality of cellular repairing mechanisms^12,25,38^.

Noticeably, cQMY has a linear relationship according to Eq. (4a), through measurable variables (wavelength and mortality). Hence, it is possible to calculate cQMY by counting dead cells after in at least two exposure times. Another way to obtain cQMY is to construct a graphic of N versus ln(t) where the slope corresponds to:

3a $${\text{k}} = {\text{slope}} = {\Phi }_{{\upmu }} {\text{E}}_{{\text{a}}} = {\Phi }_{{\upmu }} {\text{n}}_{0} {\text{hc}}/{\uplambda }$$

**Figure 2:**
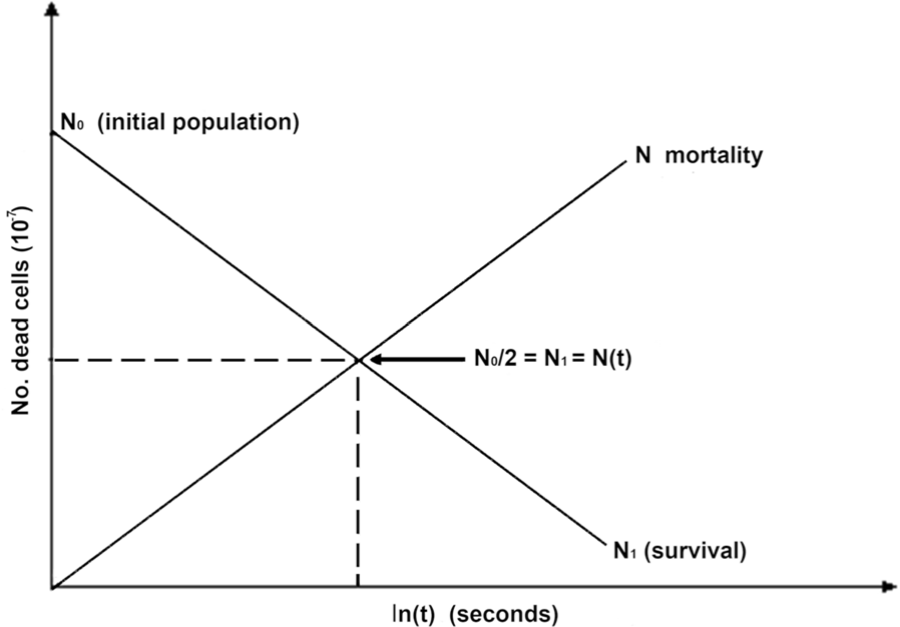
Viability and mortality curves.

On the other hand, when N_1_ is charted versus ln(t), the expected curve is a straight line passing through the ordinate-axis and moving up or down by changes in the negative slope. Such intersect corresponds to N_0_. The slope of this survival curve is associated to the target size, this is to say, to the size of the genome at a constant repairing time. In the case where values of cQMY are the same for two microorganisms, its ratio to the relative genome sizes could be calculated from each slope, thus allowing the differentiation between species (Fig. 2). However, ${\Phi }_{\mu }$ changes with the repairing capacities of each cell and as a function of the degree of damage^13,39^. It is also worth noting that microorganisms with the same quantity of DNA and similar pyrimidine/purine ratios might have different sensitivities to the lethal effects of radiation depending on its genomic sequence; therefore each species have unique mortality times.

From Fig. 2, it can be observed that the intersection point between the mortality and survival curves corresponds to an equilibrium state where the number of dead is the same as the surviving cells (N = N_1_), this point is also known as the Lethal Dose of Radiation (*LDR*_*50*_).

##### Lethal dose of radiation

LDR_50_ is defined as the exposure time needed to kill half a microbial population. Given Eq. (3) and considering that at LDR_50_ $${\text{N}} = {\raise0.7ex\hbox{${N_{0} }$} \!\mathord{\left/ {\vphantom {{N_{0} } 2}}\right.\kern-\nulldelimiterspace} \!\lower0.7ex\hbox{$2$}}$$, the intersection point using Eq. (4) is resolved as:

5 $${\text{t}} = {\text{e}}^{{{\text{N}}_{0} / \left( {2{\Phi }_{{\upmu }} {\text{ E}}_{{\text{a}}} { }} \right)}} = {\text{e}}^{{{\text{N}}_{0} / \left( {2{\text{k }}} \right)}}$$

In the special case where the population is only two microbial cells irradiated (N_0_ = 2):

5a $${\text{t}} = {\text{e}}^{{1 / {\text{k }}}}$$

from here we know that $$ln\left( t \right) = {\raise0.7ex\hbox{$1$} \!\mathord{\left/ {\vphantom {1 k}}\right.\kern-\nulldelimiterspace} \!\lower0.7ex\hbox{$k$}}$$ and this value corresponds to the minimal exposure period required to produce one dead cell, also known as Minimum Mortality Time (MMT, $${\text{t}}_{{\text{u}}}$$) :

6 $${\text{ t}}_{{\text{u}}} = {\raise0.7ex\hbox{$1$} \!\mathord{\left/ {\vphantom {1 {\text{k}}}}\right.\kern-\nulldelimiterspace} \!\lower0.7ex\hbox{${\text{k}}$}}$$

##### Minimum time of mortality

The energy recquired to produce the death of one cell is provided by the incidence of UV-C light quanta on each nitrogenous base, instead of the uniform distribution of total energy on a wide genomic area that is contemplated by the wave-like perspective of the diffusion of light over matter (assumption 2).

Cell inactivation occurs in the same time-lapse at which photoreactions are happening. This brief period is such that the sum of the times of each individual photon-base collision is equal to ${\mathrm{t}}_{\mathrm{u}}$, accounting that the repairing mechanisms are not fast enough to contain the damage.

On the other hand, we can express Eq. (6) on terms of wavelength as:

$${\text{t}}_{{\text{u}}} = \frac{{\uplambda }}{{{\Phi }_{{\upmu }} {\text{n}}_{0} {\text{hc}}}}$$

the latter equation also shows that the variation on the time for cell death on each species is directly proportional to the wavelength and conversely proportional to cQMY. It also can be observed that MMT is defined by the combination of three fundamental constants of physics with cQMY, so both variables could be considered as intrinsic biological constants, associated to the nature and composition of a genome. MMT is a crucial concept for the development of our model and deserves special description.

Mortality is frequently a polemic issue because cell death does not happen instantly and it can be described by a series of biochemical events and photochemical reactions. In here we adopted a simplified approach where MMT coincides with the formation of pyrimidine dimers in a context of insufficient repairing systems, the latter is considered as cellular inactivation.

##### Absolute death state

The absolute death of a microorganism population is such that the number of surviving cells must be N_1_ = 0, under such premise, according to Eq. (4) the time required to reach this state (T) could be expressed as:

6a $${\text{T}} = {\text{e}}^{{\left( {{\raise0.7ex\hbox{${{\text{N}}_{0} }$} \!\mathord{\left/ {\vphantom {{{\text{N}}_{0} } {\text{k}}}}\right.\kern-\nulldelimiterspace} \!\lower0.7ex\hbox{${\text{k}}$}}} \right){ }}}$$

#### Mathemathical model

##### Analytic approach

The physical model describes the survial and mortality kinetics for entire cellular populations. We then aim to mathematically derive equations from molecular events as the initial point and deducting the behavior of individual cells and populations.

In here, the total sum of accumulated photon-base collitions determining the death of a single cell is called *unitary mortality process*. The photodynamics of this process obliges to define the properties of cell populations in function of discrete variables with fixed values contained on discrete parameters as listed on Tables 2 and 3.

**Table 2:** Definition of the parameters used at physical and mathematical modeling

Parameter	Definition	Units
$\delta$	Absorbed fraction	–
l	Mean free path	cm
N*	Number of total bases per genomic volume	Bases/cm^3^
N_j_	Number of base pairs	bp
V_G_	Genomic volume	cm^3^
A_G_	Genomic area	cm^2^
Z	Effective macroscopic section (EMS)	cm^−1^
f(t_u_)	Lethal impact number symbol (LIN)	–
*Φ*_*µ*_	Cell quantum mortality yield (cQMY)	mol/erg.s
*E*_*a*_	Activation energy	Einstein, erg/mol
*N*	Number of dead cells	–
*t*	Exposure time	s
*k*	Specific speed of mortality	s^−1^
*N*_0_	Number of initial cells	–
*N*_1_	Number of surviving cells	–
*RLD*_50_	Radiation Lethal dose at 50%	s, min
*MMT*	Minimum mortality time	S
*t*_*u*_	Minimum mortality time symbol	S

(−) adimensional

**Table 3:** Physical parameters of the models (constants).

Parameter	
n_0_ = 6.0225 × 10^23^ mol^−1^	Avogadro´s constant
h = 6.6256 × 10^−27^ erg.s	Planck´s constant
c = 2.9979 × 10^10^ cm/s	Speed of light
λ = 2.537 × 10^−5^ cm	Wavelength of UV-C light lamp
V_G_ = 4.3716 × 10^−13^ cm^3^	Mean genome volume
ΔX_i_ = 5.5111 × 10^−5^ cm	Mean genome thickness
*σ = 4.9627 × 10^−16^ cm^2^	Effective impact section
N_j_ = 5.2 Mpb	Mean number of base pairs

*See the Appendix 1 derivation of EIS^22^.

#### Mean free path

In here, we used an statistical mechanical concept known as free path^40^ to define the trajectory that a photon follows from the moment of its impact on the genome until its absorption by a nitrogenous base. We assume that during its displacement, a photon moves at the speed of light (*c*) in a straight line and the mean distance of these motions is called mean free path (MFP). For photons passing without impacting any molecule, MFP would be infinite. For molecules with a big area without internal movement of photons, MFP would be zero. Actually, every nitrogenous base has a finite extension, then MFP is related to the size of the target, to the genome density and to the speed of impact. Since the photon is considered as a particle with rapid movement, it interacts with the target molecule during a short interval and the impact section depends on the speed (or energy).

To calculate the MFP in a simple manner, an area around the target could be constructed such that an absorption occurs if the trajectory of an impinging photon passes through it. This area is known as Effective Impact Section (EIS) and is represented by *σ*. A photon flux in a time *t* could traverse a volumen with a *σ* of *ct* and photons would collide with all the molecules with centers included in such volumen. For a continuos flow, the first photon covers a distance $c\Delta t_{1}$ finishing at the moment of its impact, a second photon travels $c\Delta t_{2}$, a third $c\Delta t_{3}$, and so on, in such way that the total distance is:

$${\text{c}}\Delta t_{1} + {\text{c}}\Delta t_{2} + {\text{c}}\Delta t_{3} + \cdots \; \cdots + {\text{c}}\Delta t_{i}$$

$$\Delta {\text{x}} = {\text{c}}\mathop \sum \limits_{{\text{i}}} \Delta {\text{t}}_{{\text{i}}}$$

where each time interval has a unique value.

If there are $N^{*}$ bases in a genomic volume ($V_{G}$), the number of collisions at the time *t* is the number of remaining available bases ($2N_{j}$) in the cross-section *σ*. This is the probability of occurrence of an impact, a.k.a. absorbed fraction (${\updelta }$):

$${\updelta } = {\sigma N}^{*} {\text{c}}\mathop \sum \limits_{{\text{i}}} \Delta {\text{t}}_{{\text{i}}} = {\sigma N}^{*} \Delta {\text{x}}_{{\text{i}}}$$

The mean free path ($\overline{l}$) is the average distance between impacts and then $\Delta {\text{X}}_{{\text{i}}}$ is the total distance at the total time invested on photonic impacts. The latter expression is equivalent to the genome thickness divided by the number of impacts occurring in the lapse $\sum \Delta t_{i}$:

7 $${\overline{\text{l}}} = \frac{{{\text{c}}\mathop \sum \nolimits_{{\text{i}}} \Delta {\text{t}}_{{\text{i}}} }}{{{\sigma N}^{*} {\text{c}}\mathop \sum \nolimits_{{\text{i}}} \Delta {\text{t}}_{{\text{i}}} }} = \frac{1}{{{\sigma N}^{*} }}$$

In order to find the average impact time, we considered that the time between absorption events is:

8 $$\Delta {\text{t}}_{{\text{i}}} = \frac{{\text{l}}}{{\text{c}}} = \frac{1}{{{\text{N}}^{*} {\sigma c}}}$$

The latter equation represents the excitation time^37,41^, which is an even briefer lapse than MMT. Thus the impact frequency or number of impacts in a time unit results from the reciprocal of Eq. (8):

9 $${\text{ f}} = {\text{N}}^{*} {\sigma c} = \frac{1}{{\Delta {\text{t}}_{{\text{i}}} }}{ }$$

To obtain these equations we supposed that photons reach immovable targets. On the other hand, the collision process for a photon flux could be considered as elastic collisions because several particles moving at the same speed with different paths reaching one base would made the times of consecutive hits of single photons on multiple adjacent bases.

The term $(N^{*} \sigma )$ is known as effective macroscopic section (EMS) and is represented by symbol *Z*. Considering the relationship between the time a photon requires to pass through the entire genome and the mean absorption time we have:

10 $${\updelta } = \frac{{\Delta {\text{t}}}}{{\overline{{\Delta {\text{t}}_{{\text{i}}} }} }}{ } = \frac{{\Delta {\text{x}}/{\text{c}}}}{{1/{\text{N}}^{*} {\sigma c}}}{ } = {\text{N}}^{*} {\upsigma }\Delta {\text{x}}$$

Estimation of the molecular excitation time.

Considering that EIS is $4.9627\;*\; 10^{ - 16}$ cm^2^ and substituting on Eq. (8), the average time among impacts is then:

$$1/{\text{N}}^{*} {\sigma c} = 1/\left( {2.3791\;*10^{19} \frac{bases}{{{\text{cm}}^{3} }}} \right)\left( {4.9627\;*\;10^{ - 16} \;{\text{cm}}^{2} } \right)(2.9979\;*\;10^{10} \;{\text{cm/sec}}$$)

$$1/N^{*} \sigma c = 2.8252*10^{ - 15} \;{\sec}$$

##### Estimation of the absorbed fraction

The number of available molecules in a genome could be defined in terms of its area (*A*_*G*_) and thickness (∆*x*) as:

$${\text{N}}^{*} {\text{A}}_{{\text{G}}} \Delta {\text{x}}$$

If each base has a defined EIS, the total impact area is: $\sigma (N^{*} A_{G} \Delta x)$, henceforth the probability of photon impacting the genome is a ratio of the total area or: $\sigma (N^{*} A_{G} \Delta x)/A_{G}$. To determine such ratio, we matched the equation to the absorbed fraction:

10a $${\updelta } = {\upsigma }({\text{N}}^{*} \Delta {\text{x}}) = {\text{N}}^{*} {\sigma c}\Delta {\text{t}}$$

Since $\Delta x = c\Delta t$ and considering that $N^{*} = 2N_{j} /V_{G}$, then substituting on Eq. (10) we have:

$${\updelta } = \frac{{2{\text{N}}_{{\text{j}}} {\sigma A}_{{\text{G}}} \Delta {\text{x}}}}{{{\text{V}}_{{\text{G}}} {\text{A}}_{{\text{G}}} }} = \frac{{2{\text{N}}_{{\text{j}}} {\upsigma }\Delta {\text{x}}}}{{{\text{V}}_{{\text{G}}} }}{ }$$

Also, if $A_{G} \Delta x$ is the volume occupied by the genome, then $A_{G} \Delta x/V_{G} = 1$ and the absorbed fraction is:

10b $${\updelta } = \frac{{2{\text{N}}_{{\text{j}}} {\upsigma }}}{{{\text{A}}_{{\text{G}}} }}$$

An equivalent expression is:

10c $${\updelta } = \frac{{\overline{{\Delta {\text{x}}}} }}{{{\overline{\text{l}}}}}$$

Taking a mean genome size of 5.2 millions of base pairs (Mbp)^42^ for *E. coli*, a genome volume of $4.3716\;{*}\;10^{ - 13} \;{\text{cm}}^{3}$ and a thickness of $\Delta {\text{x}} = 5.5111\;{*}\;10^{ - 5} \;{\text{cm}}$^43–45^, then substituting in Eq. (10):

$${\updelta } = \left( {2.3790{*}10^{19} \frac{{{\text{bases}}}}{{{\text{cm}}^{3} }}} \right)\left( {4.9627{*}10^{ - 16} {\text{ cm}}^{2} } \right)\left( {5.5111{*}10^{ - 5} {\text{ cm}}} \right)$$

$${\updelta } = 0.6506\;{\text{bases}};\;{\text{and}}\;{\text{Z}} = {\text{N}}^{*} {\upsigma } = 1.1806{* }10^{4} \;{\text{cm}}^{ - 1}$$

##### Lethal impact number

From Eq. (9), the product of the impact frequency times MMT is equal to the number of impacts at the time $t = t_{u}$, also denominated lethal impact number (LIN) or the number of photonic impacts received by a particular molecule in the time needed to reach mortality, and it is proportional to the number of bases in the genome (see next section).

Then:

12 $${\text{f}}\left( {{\text{t}}_{{\text{u}}} } \right) = \frac{{{\text{N}}^{*} {\sigma c}}}{k}$$

And accordingly,

13 $$k = \frac{{{\text{N}}^{*} {\sigma c}}}{{{\text{f}}\left( {{\text{t}}_{{\text{u}}} } \right)}}$$

where it can be seen that *k* is directly proportional to the number of bases in the genome volume, and also, to the EIS.

Generalization of the Unitarian equation system for mortality and impact.

The frequency of impacts occurring in the time interval necessary for the death of one cell, gives an adequate argument to know the mortality kinetics, since the change rate in a population represents a continuous function in the close interval $t_{a} \le t \le t_{b}$ and is derivable in the open interval $t_{a} < t < t_{b}$; hence, for a value of $t = \left( {t_{u} } \right)$, enclosed between $t_{a} < t < t_{b}$, is verifiable that:

14 $$\frac{{{\text{ N}}_{{\text{b}}} - {\text{N}}_{{\text{a}}} }}{{{\text{t}}_{{\text{b}}} - {\text{t}}_{{\text{a}}} }} = \frac{{{\Delta N}}}{{{\Delta t}}} = \frac{{{\text{dN}}\left( {\text{t}} \right)}}{{{\text{dt}}}}$$

then the interval between t_a_ and t_b_ constitutes a differential element of the mortality curve. Knowing that ${\text{f}}\left( {{\text{t}}_{{\text{u}}} } \right)$ happens in ${\text{ t}} = \left( {{\text{t}}_{{\text{u}}} } \right)$, then:

15 $${\text{f}}\left( {{\text{t}}_{{\text{u}}} } \right) = \frac{{{\text{N}}^{*} \sigma {\text{ct}}_{{\text{u}}} }}{{\text{t}}}$$

rearranging the equation for t_u_ = t_b_ − t_a_ and considering Eq. (14) we get the following expression:

16 $${\text{N}}_{{\text{b}}} = {\text{N}}_{{\text{a}}} + \left( {{\text{t}}_{{\text{b}}} - {\text{t}}_{{\text{a}}} } \right)\frac{{{\text{dN}}\left( {\text{t}} \right)}}{{{\text{dt}}}} = {\text{ N}}_{{\text{a}}} + \left( {{\text{t}}_{{\text{u}}} } \right)\frac{{{\text{dN}}\left( {\text{t}} \right)}}{{{\text{dt}}}}$$

Substituting in Eq. (16), from $N_{a} = 0$ until $N = N_{b} = 1$ and for the death of a single cell as a condition of Eq. (15) with t_a_ = 1, we have:

$$\frac{{{\text{dN}}\left( {\text{t}} \right)}}{{{\text{dt}}}} = \frac{{{\text{N}}^{*} {\sigma c}}}{{{\text{f}}\left( {{\text{t}}_{{\text{u}}} } \right){\text{t}}_{{\text{a}}} }} = {\text{k}}$$

This last expression is the definition of the SSM. Solving the differential equation we get the following equality for the mortality:

17 $${\text{N}} = \frac{{{\text{N}}^{*} {\sigma c}}}{{{\text{f}}\left( {{\text{t}}_{{\text{u}}} } \right)}}\ln \left( {\text{t}} \right)$$

On the other hand, the number of surviving microorganisms is:

18 $${\text{N}}_{1} = - { }\frac{{{\text{N}}^{*} {\sigma c}}}{{{\text{f}}\left( {{\text{t}}_{{\text{u}}} } \right)}}\ln \left( {\text{t}} \right) + {\text{N}}_{0} { }$$

Equations (17) and (18) are analogue expressions to Eqs. (3) and (4), of our physical model. From Eq. (16) we can deduct that the MMT is given by:

$$\frac{{\left( {1 - 0} \right){\text{f}}\left( {{\text{t}}_{{\text{u}}} } \right)\left( 1 \right)}}{{{\text{N}}^{*} {\sigma c}}} = \left( {{\text{t}}_{{\text{b}}} - {\text{t}}_{{\text{a}}} } \right) = {\text{t}}_{{\text{u}}} { }$$

and then:

19 $${\text{ t}}_{{\text{u}}} = \frac{{{\text{f}}\left( {{\text{t}}_{{\text{u}}} } \right)}}{{{\text{N}}^{*} {\sigma c}}} = {\text{f}}\left( {{\text{t}}_{{\text{u}}} } \right)\Delta \overline{{{\text{t}}_{{\text{i}}} }}$$

The latter equation indicates that the sum of the individual collisions corresponds to the MMT or to the number of impacted bases in the same time.

Under this argument and from Eq. (16), again is possible to deduct the unitary equation of mortality and impact:

$${\text{N}}_{{\text{b}}} = {\text{N}}_{{\text{a}}} + \left( {{\text{t}}_{{\text{b}}} - {\text{t}}_{{\text{a}}} } \right)\frac{{{\text{N}}^{*} {\sigma c}}}{{{\text{f}}\left( {{\text{t}}_{{\text{u}}} } \right){\text{t}}_{{\text{a}}} }}$$

when ${\text{N}} = {\text{ N}}_{{\text{b}}} = 1$, because ${\text{N}}_{{\text{a}}} = 0$, then:

$${\text{N}} = \frac{{{\text{N}}^{*} {\sigma c}}}{{{\text{f}}\left( {{\text{t}}_{{\text{u}}} } \right){\text{t}}_{{\text{a}}} }}{\text{t}}_{{\text{u}}} = 1$$

Replacing ${\text{t}}_{{\text{u}}}$ for $1/{\text{k}}$ and ${\text{t}}_{{\text{a}}} = 1$

20 $${\text{ N}} = \frac{{{\text{N}}^{*} {\sigma c}}}{{{\text{f}}\left( {{\text{t}}_{{\text{u}}} } \right){\text{k}}}} = 1$$

This last equation is a particular case of Eq. (17) which shows, along with Eq. (18), that SSM is directly associated to the genome size, EIS and *c* but conversely to the number of impacts received by each base in the MMT. The total number of bases is constant and characteristic of each species of microorganism **(**see Appendix 2 for derivation).

### Predictions and theoretical results

The results obtained by the developed equations in the mathematical model allow us to estimate, under the proportionality hypothesis of assumption 7, theoretical values for the studied parameters (SSM, MMT and cQMY). Data on the number of base pairs associated to each species and genus were obtained from the literature; these are related to the dimension of their nucleoid and to the bacterial size. Other values are related to the corpuscular nature of light.

Given the Eq. (17) for cellular mortality, where the value for the SSM is given by Eq. (13), knowing the implicit parameters and considering that $\overline{{N_{J} }} \propto f\left( {t_{u} } \right)$ or $\overline{{N_{J} }} \approx f\left( {t_{u} } \right)$, we have:

13a $${\overline{\text{k}}} \approx \frac{{2{\sigma c}}}{{\overline{{{\text{V}}_{{\text{G}}} }} }}$$

where $\overline{{V_{G} }}$ is the average of genome volume^44–47^. Substituting the known values for *E. coli,* we get:

$$\overline{k} \approx \frac{{2{\sigma c}}}{{\overline{{{\text{V}}_{{\text{G}}} }} }} \approx \frac{{2\left( {4.9627\;{*}\;10^{ - 16} \;{\text{cm}}^{2} } \right)\left( {2.9979\;{*}\;10^{10} \;{\text{cm/s}}} \right)}}{{4.3716\;{*}\;10^{ - 13} \;{\text{cm}}^{3} }}$$

$$\overline{k} \approx 6.8065\;*\;10^{7} \;{\text{s}}^{ - 1}$$

If MMT is the reciprocal of *k*, then we have a mean value of:

$$\overline{{{\text{t}}_{{\text{u}}} }} = {\raise0.7ex\hbox{$1$} \!\mathord{\left/ {\vphantom {1 k}}\right.\kern-\nulldelimiterspace} \!\lower0.7ex\hbox{$k$}} \approx 1.4691\;{* }\;10^{ - 8} \;{\text{s}}$$

In the molecular context, the energy distribution in the excited bases or “*hot bases*” is widely enriched as compared to the molecules in basal states; this is, electronically, vibrationally and rotationally. According to our estimations, the excited molecules will have a half-life of $10^{ - 8}$s^37,41^, and its energy would be either transferred to the adjacent pyrimidine to form a dimer or released with the consequent recovery of the basal state. This process occurs in the whole genome matrix, and could inactivate the cell if the UV-C light stimuli are constant and the repairing mechanisms are not sufficient.

The mean cQMY was calculated from Eqs. (13a) and (3a):

21 $$\bar{\Phi }_{{\upmu }} \approx \frac{{2\bar{\sigma }\lambda }}{{{\text{V}}_{{\text{G}}} {\text{n}}_{0} {\text{h}}}}$$

Using the values of the proportionality hypothesis and the result for the SSM, we can calculate the cQMY as:

$${\overline{\Phi }}_{\mu } \approx \frac{{2\left( {4.9627\;*\;10^{ - 16} \;{\text{cm}}^{2} } \right)\left( {2.537\;*\;10^{ - 5} \;{\text{cm}}} \right)}}{{\left( {4.3716\;*\;10^{ - 13} \;{\text{cm}}^{3} } \right)\left( {6.0225\;*\;10^{23} \;{\text{mol}}^{ - 1} } \right)\left( {6.6256\;*\;10^{ - 27} \;{\text{erg}}.{\text{s}}} \right)}}$$

$${\overline{\Phi }}_{\mu } \approx 1.4435\;*\;10^{ - 5} \;{\text{mol}}/{\text{erg}}.{\text{s}}$$

Noticeable, data is on the range of magnitude orders of those previously reported by others authors^23,26,48^.

### Experimental results

We statistically analyzed the data obtained in the laboratory to graph the natural logarithm of exposure time and the mortality/survival of the exposed population. To simultaneously proof the results obtained through the models, regression coefficients and the intercepts were determined.

The first step was to elaborate a plot using the CFU/ml counted for each strain to decide which was the best model describing the survival of *E. coli* under UV-C light exposure at several times (Fig. 2A). Data were distributed in a logarithmic curve (survival decreases with exposure time), with $N_{0}$ and *k* as constants for the initial number of microorganisms ($t = 0$). We use a regression model for the best fit, to establish the relationship between the variables and to check the theoretical prediction obtained from the mathematical models.

**Figure 3:**
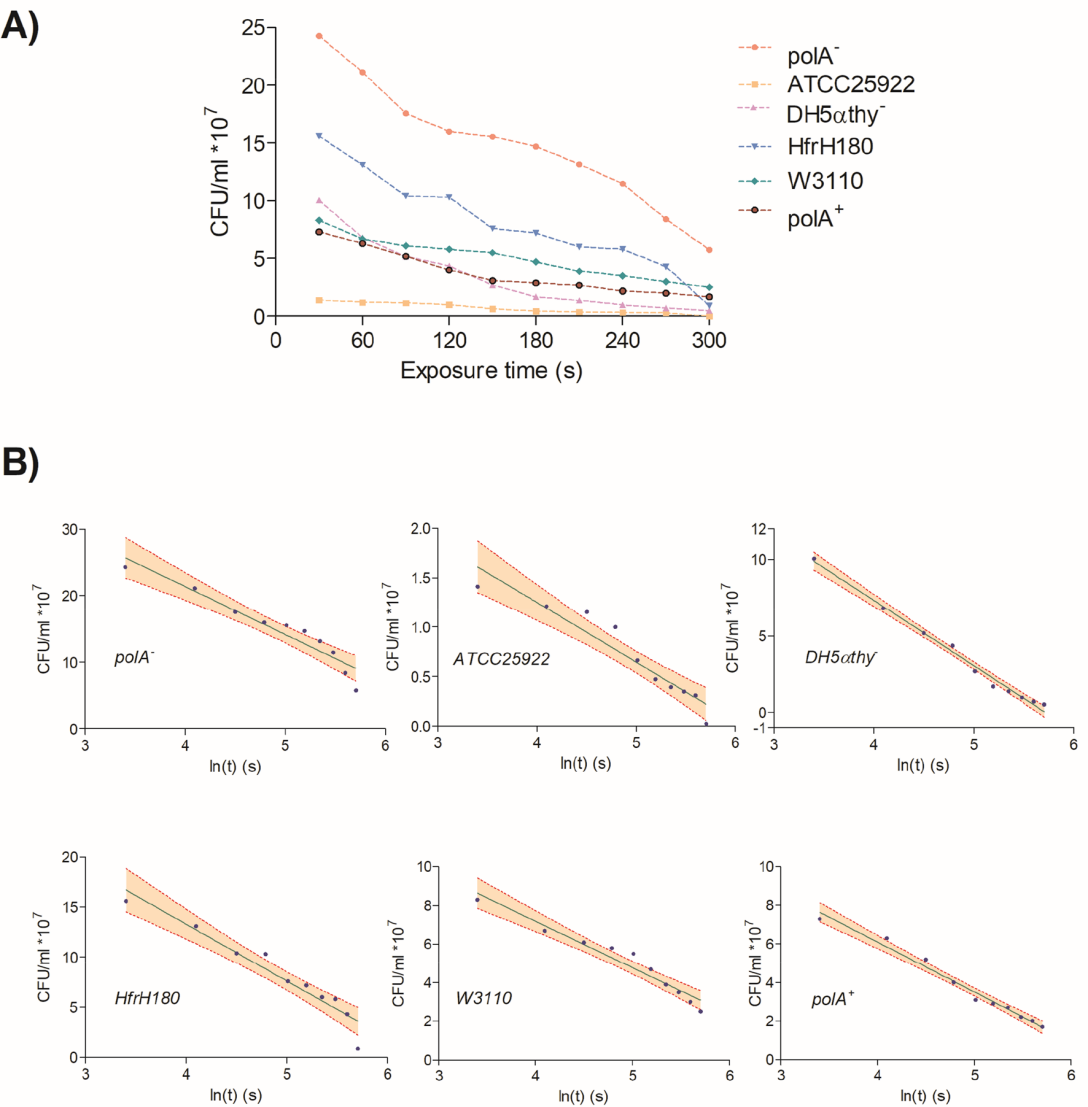
Survival curves of E coli strains. (**a**) Representation of survival of all used strains. (**b**) Dispersion plots with fiduciary limits (red lines) and confidence intervals (fawn colored area).

It can be seen from the Fig. 3A that data had a trend towards a linear behavior, with a slope or regression coefficient corresponding to the SSM. After constructing the confidence interval, it can be seen that 90% of the data were included inside the ranges (Fig. 3B).

**Figure 4:**
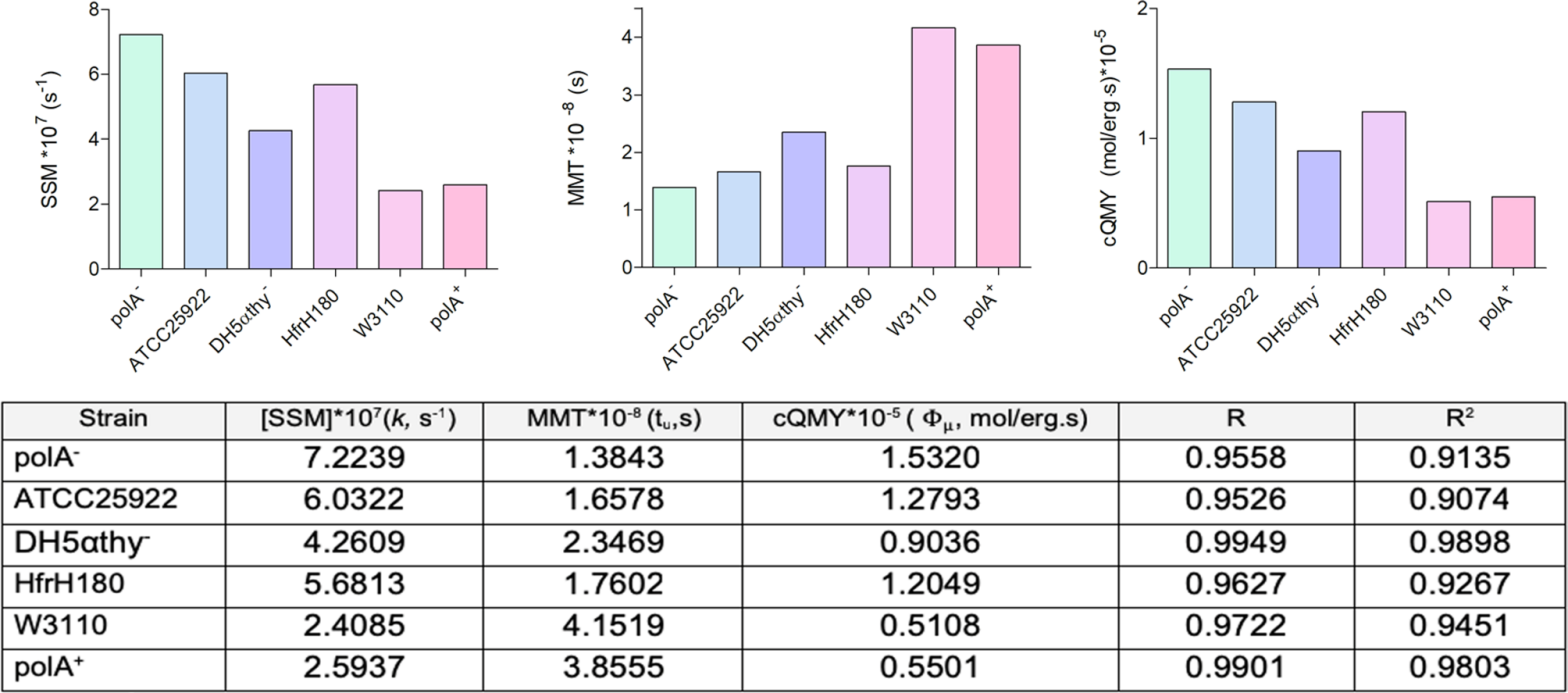
Graphical representation and calculated values of the measured parameters. SSM and R are presented in absolute values. R: correlation coefficient, R^2^: determination coefficient.

Then, cQMYs were estimated using the regression coefficients (Fig. 4). It is also shown a complementarity between the variables, for instance, polA^-^ strain (light green bar) had the highest SSM meaning that it dies with a shorter exposure time (lower MMT) and it had a high susceptibility to UV-C light exposure (high cQMY).

To compare the observed means of SSM against the estimated values, a t-Student test was performed considering 0.97 of confidence, 0.03 as significance level. The results showed *p* ≥ 0.045 (data not shown). Hence, values had not a statistically significant difference.

#### Proportionality hypothesis

The quantity and distribution of the nitrogenous bases and the genomic volume are intrinsic features of each genus and species and these parameters are determinant when calculating the SSM, because it changes inversely with V_G_. In here, we hypothesized that the number of photons impacting a genomic matrix is proportional to the number of base pairs and that the coefficient of these two values must be nearly equal to one^23,28^.

Then, using Eqs. (2a), (13) and (13a), we derived an equation to test the validity of the proportionality hypothesis:

22 $$\frac{{{\text{N}}_{{\text{j}}} }}{{{\text{f}}\left( {{\text{t}}_{{\text{u}}} } \right)}} = \frac{{\Phi _{{\upmu }} {\text{n}}_{0} {\text{hV}}_{{\text{G}}} }}{{2\sigma \lambda }}$$

In some of our experimental observations this coefficient resulted in values close to one, except for W3110 and polA^+^ strains. To compare other proportionality coefficients, Eq. (22) was applied to data previously reported in the literature (Table 4).

**Table 4:** Assumption (7) for Eq. (22) in experimental and other *E. coli* strains.

Strain	Wavelength (λ, nm)	Assumption 7; Eq. (22)	$D_{p}$	Reference
*Experimental results*				
polA^-^	253.7	1.0613	65,274	–
ATCC25922	253.7	0.8862	78,168	–
DH5αthy^-^	253.7	0.6260	110,664	–
HfrH180	253.7	0.8346	82,994	–
W3110	253.7	0.3538	195,771	–
polA^+^	253.7	0.3810	181,798	–
*Referenced values*				
K12 (1981)	260	1.0578	65,488	Jagger^26^
–	254	1.4076	49,213	Harm^48^
IFO 3,301	254	2.7709	25,000	Oguma^12^
K12 (wild type)	254	4.4335	15,625	Howard-Flanders^39^

Nonetheless, Eq. (22) does not describe the number of photons necessary for the formation of pyrimidine dimers; to that end, we obtained the following expressions:

23 $$\Phi _{\upmu } = {\text{D}}^{ \wedge } {\text{p/}}\gamma _{{{\text{abs}}}}$$

24 $${\text{D}}_{{\text{p}}} = 1/\Phi _{\upmu } = \gamma _{{{\text{abs}}}} /{\text{D}}^{ \wedge } {\text{p}}$$

where ${\text{D}}_{{\text{p}}}$ is the number of formed pyrimidine dimers, $D_{p}$ is the number of photons necessary for the formation of a single dimer and $\gamma_{abs}$ is the number of absorbed photons per cell. The reciprocal values of cQMY are shown in Table 4, confirming postulate 7 and the multi-impact nature of UV-C light propagation on DNA.

## Discussion

According to our equation system, the determination of cQMY, SSM, MMT and ${\text{D}}_{{\text{p}}}$ are crucial when describing UV-C light incidence on a genome. These four parameters are sensible indicators of the degree of damage of an exposed genomic matrix.

Beggs^23^ reported that Φ_µ_ might be ranging from 10^–6^ to 10^–5^ and in Table 4 we enlisted other cQMYs^12,26,39,48^. Noticeably, the yields calculated experimentally are on the same order of magnitude, showing a high level of fitness in our equations. The proportionality coefficient of Eq. (22), varies from 1.03 to 4.00 (Table 4), we attribute these discrepancies to the diversity of methodologies used by each author.

The mathematical model predicted a value of SSM of 6.8 * 10^7^ s^-1^ and the experimental results show a range of 2–7 * 10^7^ s^-1^, without a significant difference between both values (p = 0.045). In regard to MMT, the experimental results are within the range of the predicted value (1.4 * 10^–8^ s) (Fig. 3) and coincide with other mortality times previously reported for the duration of the excitation state. We determined that photons could lead a base into its excitation state at a rather fast time (2.8252 * 10^–15^ s, λ = 253.7 nm), which is also in accordance with other reports^35^.

Another useful data in the simulation of the process is the absorbed fraction (${\updelta }$). In our experiments, ${\updelta } = 0.65$, implying that approximately 35% of an *E. coli* genome is not hit.

Variations on this value are due to the genomic area, base-distribution and sequence configuration of each strain.

We used 5.2 Mbp as the mean genomic size according to the GENOME database for *E. coli*^42^, although other authors use 4.6 Mbp as the mean size (4′639,221 bases)^13,49^, and even in the literature, values ranged from 4.6 to 5.5 Mbp; nonetheless, by substituting these alternative values the results are approximate to those previously reported.

In regard to the physical model, although single impact developments are used in other biological models of UV-inactivation, they do not completely explain the dynamics of the process^23^, nor does the models with a continuous light diffusion approach (undulatory behavior). We considered that the corpuscular approach is more adequate since a quantum model results useful to explain why several photons are required to cause a pyrimidic dimerization. Besides, the repairing mechanisms play an important role because a cell with a low repairing rate could not overcome dimer formation and it might be unable to maintain its integrity. cQMY is directly associated with the wavelength of the incident light and to the genome size, which make it an appropriate measure of the effectiveness of a photochemical reaction (Eq. 21) and could be considered as a biological constant, analogous to the quantum yield. Our results are mutually compatible with the unitary equations of mortality and impact and in agreement with the experimental values reported by Kumiko et al. of 4 * 10^–5^ for a genome size of 4.6 Mpb and the 6.4 * 10^–5^ reported by Howard-Flanders et al.^26,39,48^.

### Sensitivity

We showed that SSM changes directly with cQMY and conversely with MMT. Nonetheless, the variability of data between each *E. coli* strain is related to the characteristics of each genome and/or its repairing mechanisms. Pyrimidine dimers are eliminated by the nucleases coded in *uvrABC* and *PolA* genes. When the filling of gaps is produced after replication, DNA repairing could be done by recombination with an undamaged parallel strain by means of Rec proteins, particularly by RecA^50^. This protein is also necessary for the expression of the SOS regulon, because it promotes the proteolysis of the repressor (LexA) hence allowing the expression of *umuCD* genes^51^. On the other hand, it is known that deletion-insertion mutants of the DNA helicase II (*uvrD*) are UV-C light sensitive due to the high variability of its allelic genotypes^52^.

In here, the elimination genotypes of each strain produce different values for MMT, cQMY and SSM. The strain HfrH180, which does not have deficiencies on any DNA repairment system^53,54^ was moderately sensitive to UV-C light effects.

PolA^−^ strain presented a higher sensitivity to the UV-C light exposure compared to PolA^+^, probably due to its lack of the polymerase. In fact, the pair of PolA^−^/PolA^+^ strains is widely used on the study of geno-lethal effects of diverse substances on bacterial test panels^55^.

Youngs et al.^56^ showed that a higher sensitivity to UV light was presented by strains with a *recA*^*−*^ genotype, being the strains *recA*^*−*^/*uvrB*- the most susceptible. In here, the strain DH5αthy^*−*^ was less sensitive to UV-C light than HfrH180 although it does not have mutations on the uvrABC system. The ATCC25922 strain had a relatively high sensitivity only below polA^*−*^, which was unexpected, although, it partially could be explained because this strain is intrinsically sensitive to lethal agents. In fact, ATCC25922 is recommended by the CLSI as a reference microorganism to evaluate antimicrobial sensitivity. Finally, *E. coli* W3110, previously reported as UV-sensitive and with a pyrimidine auxotrophy, had low SSM but a still measurable mortality.

### Regression, correlation and determination

The regression analysis showed a strong trend towards a straight line on the dispersion diagram [ln(t) vs N, Fig. 2B] and the regression coefficients were in agreement with the theoretical calculations (SSM, *k*). The fact that the data behavior was not accurately predicted for a sample of the variables [(ln(t_i_),N_j_)_i,j_] depends on the experimental variability and the complex biological nature of test system. Let us also recall that we assume that the number of surviving or dead microorganisms is a random variable, which depends on the exposure time, that it has homogeneous variances, independence of N and N_1_ values and a normal distribution. The proposed models are adequate to describe the travel of UV-C light in a genomic environment in a more complete and real manner.

Regarding the correlation coefficients, the points on each strain showed a marked association between both variables [ln(t), N] within the interval 0.9526* ≤ R ≤ *0.9949*.* Hence, there is good evidence that the variable *ln(t)* contributes to explaining *N* and *N*_1_.

The values for the determination coefficients had a high goodness of fit to the projected curve because the explained fractions are in the interval of 0.9074* ≤ R*^2^* ≤ *0.9898, revealing a cause-effect relationship between the variables.

## Conclusions

The quantum depiction proposed in these models was useful to prove that the distribution of the activation energy from UV-C light through the genome is discontinuous. On the other hand, these are multiple-impact mechanistic models that allowed the calculation of mean population values from individual effects at the photon-molecule interaction. Finally, other population kinetics of mortality and survival could be simulated by taking into account the wavelength of the lamp used, the volume and base distribution in the genome of the microorganism in study and by contrasting data with simple viable counts measures from the resulting cultures.

## Perspectives

We believe that is necessary to consider the DNA repairing mechanisms to extend the parameters involved, then to improve the mathematical modeling to include eukaryotic cells or tissues. Additionally, antimutagenic capacity of candidate compounds projected to be used as protection from the effects of UV light, can be further studied since the equations describe the mechanisms associated with damage to nucleic acids and allows its quantization. cQMY, SSM and MMT could be used as classification parameters for the mutagenic/antimutagenic potency of virtually any molecule; this is to say, to establish the grade (low, mild, high or very high) on which a substance will damage/protect a cell from UV light effects.

The system presented in here, could also be extended to other wavelengths by performing the pertinent adjustments for other affected biomolecules (proteins, lipids, carbohydrate, etc.). On the other hand, in eukaryotic cells it could be applied in tumoral tissue cells, in radiotherapy assays and in carcinogenesis animal models. Alternatively, the fitness of models could be challenged by adding molecules with known photonic absorption (molecular actinometers) to improve their accuracy.

## Supplementary information

Supplementary file1 (DOCX 4132 kb)

Supplementary file2 (DOCX 14854 kb)
