# Supplementary material for: Simplified modeling of E. coli mortality after genome damage induced by UV-C light exposure
Source: Sci Rep. 2020 Jul 9;10:11240. doi: 10.1038/s41598-020-67838-1 (PMC7347587; doi:10.1038/s41598-020-67838-1)
Supplement: Supplementary file 1 — Supplementary file1 (DOCX 4132 kb) [file 41598_2020_67838_MOESM1_ESM.docx]

**APPENDIX 1**

*Effective impact section*


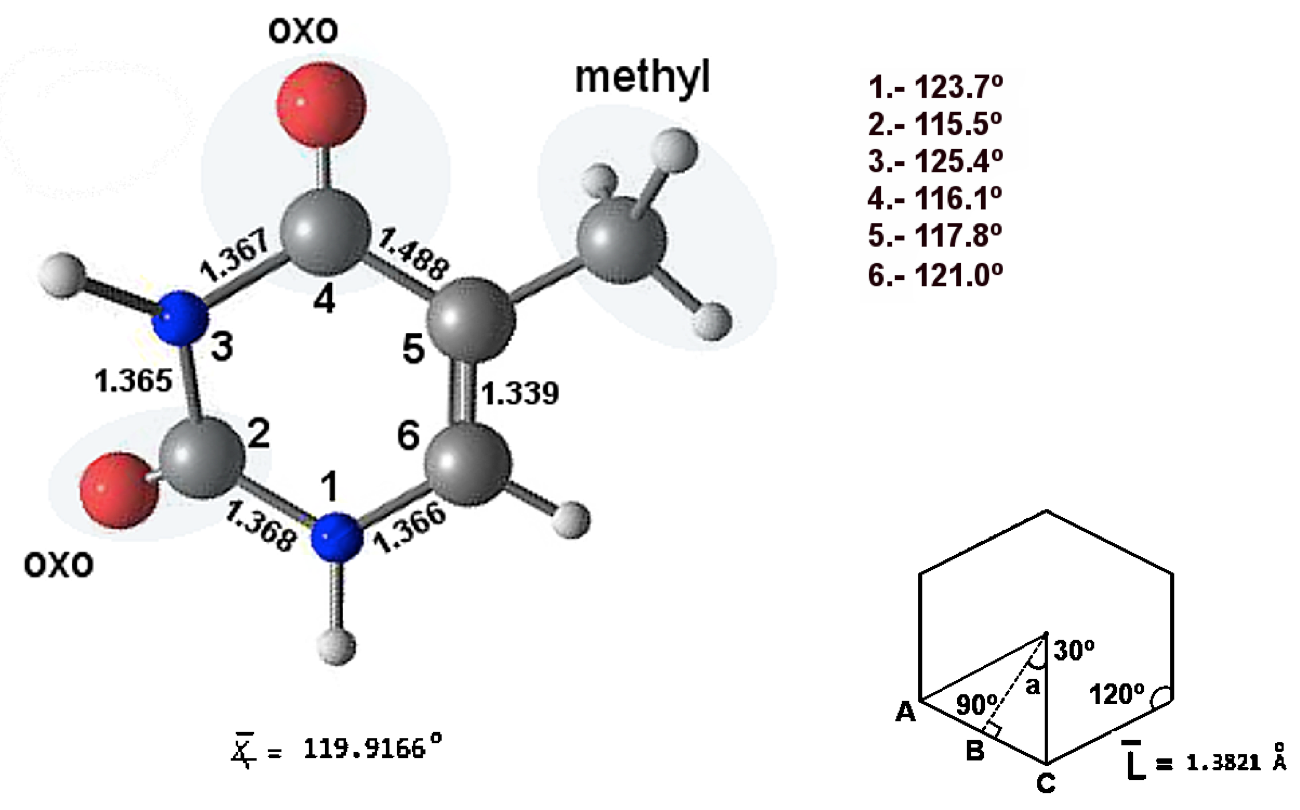
Thymine bases can be assumed as plane molecules that, in their most stable structure, have internal angles around 115.5° and 125.4°^21^. The C=C link is a short double bond of approximately 1.339 Å in length. The C-C simple bonds are long bonds with a length of approximately 1.488 Å. The bonds C-N next to an *oxo* group are 1.365-1.368 Å and for the C-N free bond is 1.366 Å.

*Figure A1. Intramolecular distances and angles in pyrimidinic rings*

Considering a hexagonal geometry, we have the following expression:

$\sigma=\left( 3/2 \right)L^{2}\cot30^{\circ}$ (A)

Equivalent expressions are:

$\sigma={L^{2}(3\surd3)}/\bar{2}$ (B)

$\sigma={3L^{2}}/2\tan30^{\circ}$ (C)

Replacing the mean values of the bonds and angles on Eqn.(B), we estimated the EIS as:

$$\sigma=\frac{\left( 3 \right)\left( 1.7320 \right){(1.3821 X {10}^{-8} cm)}^{2}}{2}=4.9627 X {10}^{-16} {cm}^{2}$$
