# Supplementary material for: Simplified modeling of E. coli mortality after genome damage induced by UV-C light exposure
Source: Sci Rep. 2020 Jul 9;10:11240. doi: 10.1038/s41598-020-67838-1 (PMC7347587; doi:10.1038/s41598-020-67838-1)
Supplement: Supplementary file 2 — Supplementary file2 (DOCX 14854 kb) [file 41598_2020_67838_MOESM2_ESM.docx]

**APPENDIX 2**

*Mean value theorem for MMT*

The mortality curve is a continuos function of the exposure time, hence there is a measurable value of time for $t=t_{u}$ corresponding to the reciprocal of its derivative.

If N is a continuous function of t at the closed interval $t_{a}\leq t\leq t_{b}$ and is differentiable at the open interval $t_{a}<t<t_{b}$, then there is at least one value of$t_{u}$ comprehended between $t_{a}$ and $t_{b}$ at which:

$$\frac{N_{b}-N_{a}}{t_{b}-t_{a}}=\frac{dN\left( t \right)}{dt}$$

This is to say, if $P_{1}$ and $P_{2}$ are two points at the mortality curve, there is at least one point between $P_{1}$ and $P_{2}$ whithin a line tangential to the curve that also is parallel to the rect $P_{1}P_{2}$ (figure A2)


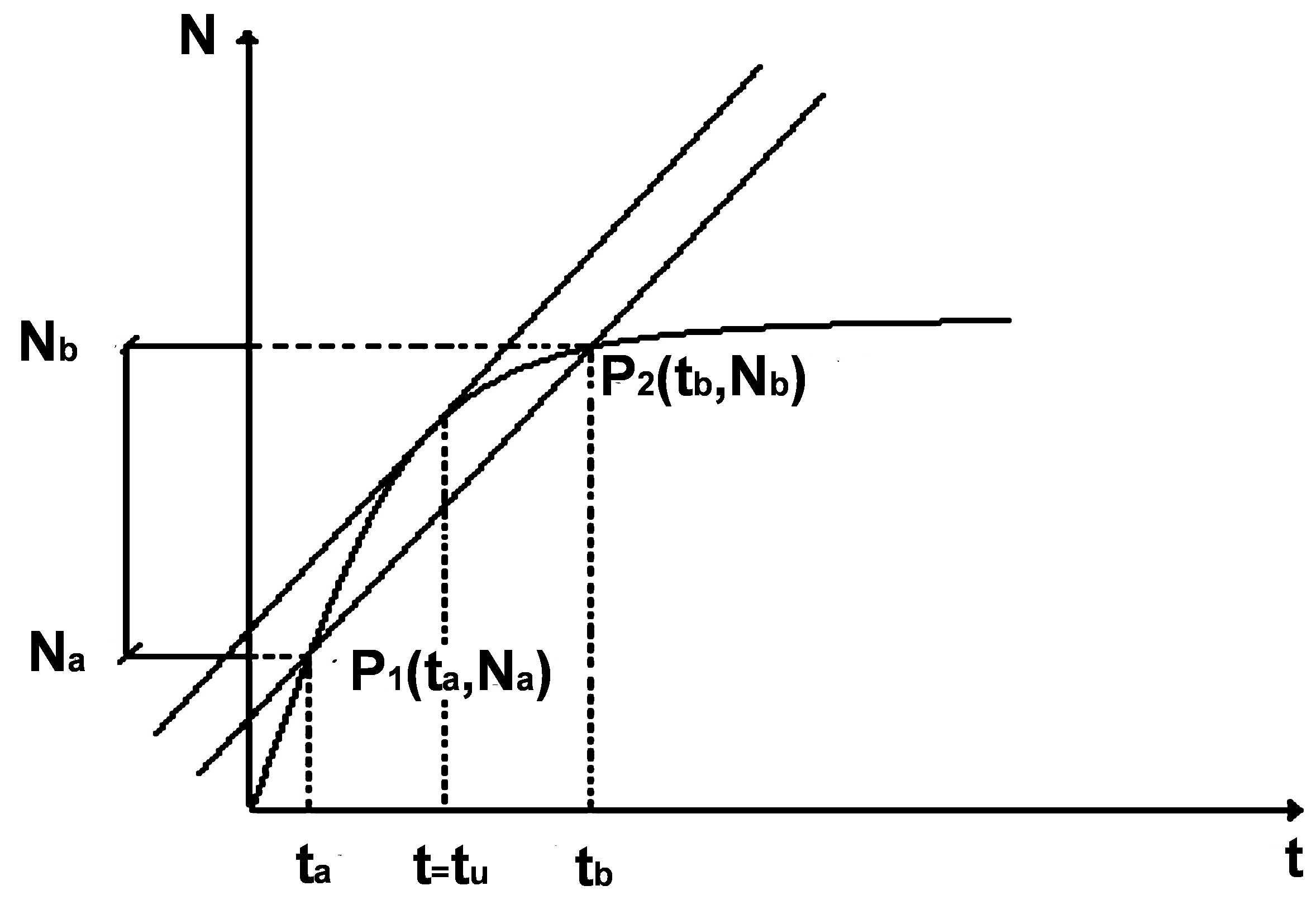


*Figure A2. Mortality curve*

The latter equation also admits the following expression:

$$N_{b}=N_{a}+(t_{b}-t_{a})\frac{d}{dt}N(t)$$

the equation for the secant of $P_{1}P_{2}$ is $N=N_{b}+k(t-t_{b})$ where $k=\frac{N_{b}-N_{a}}{t_{b}-t_{a}}$

the distance $t_{b}-t_{a}$ that is vertical to the secant of the curve is:

$$F\left( t \right)=N\left( t \right)-N_{b}-k(t-t_{b})$$

$F\left( t \right)$ satisfies the conditions for the theorem of Rolle at which $N_{a}=N_{b}=0$, where there is at least a value of t between $t_{a}{\mathrm{and}t}_{b}$ where $\frac{d}{dt}N\left( t \right)=0$, this is to say, there is a point where the tangent is parallel to the t axis.

Then, considering the interval $t_{a}< t <t_{b}$ at which N=1, according to the mean value theorem, and based on Eqn.(3), we have:

$$\frac{d}{dt}N\left( t \right)=\Phi_{\mu}E_{a}\frac{d}{dt}\ln t$$

hence:

$\frac{d}{dt}N\left( t \right)=\Phi_{\mu}E_{a}(\frac{1}{t})$; where $t=t_{a}$

If $N_{b}=1$, $N_{a}=0$ for $t_{a}=1$, then:

$$1=0+(t_{b}-t_{a})\frac{\Phi_{\mu}E_{a}}{1}$$

thus:

$$\Delta t=\left( t_{b}-t_{a} \right)=\frac{1}{\Phi_{\mu}E_{a}}=\frac{1}{k}$$

This equation permited the construction of the mathematical model for viability and mortality described in the main text. It also permited to understand the unitary impact processes that occur at the cell genome, besides it further helps to explain that the total sum of individual events of accumulated photon-molecule colisions are the MMT.
